# The Relation of the Milk Supply to Infant Disease

**Published:** 1915-06

**Authors:** M. M. Carrick


### The Relation of the Milk Supply to Infant Disease.

BY M. M. CARRICK, M. D.

The United States Census Bureau estimates that 300,000 babies less than one year old died last year in this country, and it is authoritatively stated that at least half of these deaths were needless. That there is a serious relation between this death rate and our milk supply, we doctors know only too well. That is why we are making such a desperate plea for a square deal for his majesty —the baby.

The New York Public Health Council recently issued a sanitary code dealing with milk and cream, which for the first time gives to the cities and villages of New York State a simple and uniform system of dairy regulation. According to this code, the local health officer must inspect every dairy farm at least once a year and score its sanitary condition on a standard score card. Under the code, he shall not grant a permit unless he finds the premises clean and sanitary, and unless the farm or dairy scores at least forty out of a possible hundred points on the score card.

Another important point is that the milk from farms which pass the test is furthermore to be graded in one of seven classes, and labeled with its proper grade. The highest grade of milk is "certified." This means that it is "certified" as coming up to the regulation of a recognized medical milk commission. Then follows three grades: A, B, C. In each of these grades is a raw milk class, and a pasteurized milk class.

For instance, the code points out an important fact that so-called pasteurized milk is often a misnomer; that it is only heated for a few seconds and the disease germs are not destroyed. The code, therefore, specifically states just how long milk must be heated to be pasteurized, giving 145 degrees Fahrenheit for thirty minutes, at least, as the safe rule.

Among the other tests, in the different grades, it is specified that milk must come from cows tested by the tuberculin method at least once a year. The dairy score, and the maximum bacteria count, are also fixed in each of these grades of milk and cream.

These grades are labeled in different colors—black, green, red. When all this system is perfected the buyer will know from the label exactly what he is getting, and the sanitary dairyman will receive the recognition that he deserves.

Already war has been declared on the skimmed milk menace. Unsuspecting mothers who have hitherto purchased this by-product from containers in corner groceries and dairies are being warned that such milk is not for infant-feeding.

Here are a few reasons why milk should be pasteurized:

First, because raw milk causes untold infant deaths. I would say 25 per cent under five years of age are thus caused.

More children die from intestinal diseases than from other causes. Children's food is chiefly milk.

Dirt and bacteria harmless to adults irritates the intestines of children. Pasteurization kills 90 per cent of the bacteria in milk.

Raw milk causes septic sore throat. This is a violent form of tonsilitis. It is often followed by acute articular rheumatism, erysipelas, peritonitis, endocarditis, and other serious inflammations.

Boston had 1043 cases from one raw-milk supply. Chicago had 10,000 cases from raw-milk supply. Baltimore had 602 cases.

The bacteria found in the udders of cows closely resembles the bacteria found in these sore throats.

Pasteurization kills the bacteria producing septic sore throats. No epidemic has ever been traced to pasteurized milk. When clean milk cannot be had; therefore, always heat that which is used. Bad milk may look clean and may taste sweet and smell sweet. Disease germs do not make their presence known by ordinary tests.

It will be wise to send to the chief of the Children's Bureau, Washington, D. C., for free directions for pasteurizing milk. A monograph on "Infant Feeding" may also be had for the asking. All these helps will go a long way toward giving the baby a square deal.

